# The Dual Pathways Hypothesis of Incel Harm: A Model of Harmful Attitudes and Beliefs Among Involuntary Celibates

**DOI:** 10.1007/s10508-025-03161-y

**Published:** 2025-05-21

**Authors:** William Costello, Joe Whittaker, Andrew G. Thomas

**Affiliations:** 1 Department of Psychology, University of Texas at Austin, Austin, TX USA; 2 School of Psychology, Swansea University, Swansea, SA2 8PP UK; 3 Department of Criminology, Sociology, and Social Policy School of Social Sciences, Swansea University, Swansea, UK

**Keywords:** Involuntary celibates, Incels, Misogyny, Mental health, Singlehood, Dark Triad, Autism

## Abstract

Incels (involuntary celibates) are an online subculture of men who form their identity around a perceived inability to form sexual or romantic relationships. This community operates almost exclusively online, often serving as an outlet for misogynistic hostility. Concerns about violence from incels have positioned them as a growing (inter)national security threat. This study, the largest primary investigation of incel harmful attitudes and beliefs to date (*N* = 561), recruited participants from the USA and the UK with a mean age of 26 years. Consistent with previous research, the sample showed ethnic and political diversity, poor mental health, high levels of suicidal ideation, and autistic traits. Using a 3N (needs, networks, and narratives) informed theoretical framework, our pathway analysis revealed that poor mental health and ideological adherence were twice as predictive of harmful attitudes and beliefs (e.g., displaced aggression, hostile sexism, and justification of violence) compared to networking, with a bidirectional effect between poor mental health and ideology. We also found two distinct indirect pathways to harmful attitudes and beliefs among incels: one involving experiential vulnerabilities (e.g., autism traits, low mate value, and histories of bullying and abuse) and the other rooted in dispositional traits (e.g., the dark triad and right-wing political orientation), leading us to propose the dual pathways hypothesis of incel harm. These findings suggest that interventions targeting mental health and ideology may be more effective than those focusing solely on online networking. Implications for intervention strategies, informed by these theoretical models, are discussed, including date coaching, therapy and role modeling from former incels. As issues of extremism, online radicalization, and mental health grow in importance, these insights are vital for policymakers, educators, journalists, and others addressing the challenges incels face and represent in society.

**Supplementary Information:**

The online version contains supplementary material available at 10.1007/s10508-025-03161-y.

## Introduction

Incels (involuntary celibates) are an online subculture of men who identify with their perceived inability to establish sexual relationships. The community is an outlet for misogynistic hostility (Speckhard et al., 2021), and incels’ ideology includes the belief that most women are attracted to a small number of men, who monopolize sexual encounters with women (Baselice, 2024). Central to the incel belief system is the “black-pill” philosophy that there is nothing they can do to improve their romantic prospects (Glace et al., 2021).

In recent years, incels have gained prominence in national and international security discussions due to a small number of ideologically motivated spree-killers (Costello & Buss, 2023). The community has been labeled as an emerging domestic terrorism concern by counterterrorism authorities in Canada, the USA, and the UK (Canadian Security Intelligence Service, 2024; Hoffman et al., 2020; Law Commission, 2021). These concerns align with broader findings that misogyny serves as a shared psychological mechanism underpinning various forms of male violence, including violent extremism, interpersonal violence, and violence against women (Rottweiler et al., 2023).

### Prior Research on Incels

Most incel research is limited to online content analysis (e.g., Helm et al., 2022), which overlooks the fact that incels’ online behaviors are often performatively antagonistic (Daly & Nichols, 2023) and overrepresent the views of a vocal minority (Jaki et al., 2019). Recently, primary quantitative studies have emerged, focusing on ideology and the prevalence of incels’ mental illness diagnoses (e.g., Moskalenko et al., 2022). Involuntary singlehood is linked to low emotional wellbeing (Apostolou et al., 2024), and incels report high levels of depression, anxiety, and loneliness (see Costello et al., 2024a for a review). However, this research has mostly been descriptive, illustrating heterogeneity among incels. Few studies exceed sample sizes of *N* = 250, limiting the capacity to rigorously explore or predict which subgroups of incels have a propensity for harmful attitudes and beliefs (Schönbrodt & Perugini, 2013). This relative paucity of research has led to a call for a greater focus on gaining access to primary data to better expand our knowledge of the incel subculture (Hart & Huber, 2023).

### Current Study and Theoretical Framework

This study investigates the psychological, ideological, and networking factors that predict harmful attitudes and beliefs among self-identified incels. We draw upon the 3N theory of radicalization (Kruglanski et al., 2019, 2022; Webber & Kruglanski, 2017), which conceptualizes radicalization through three interconnected factors: Needs, Narrative, and Network. Needs reflect an individual's psychological drivers, such as mental health issues or a need for significance. Narrative encompasses the ideological beliefs that justify grievances and violence, and Network refers to the social connections that reinforce these narratives.

The 3N model has been validated in several contexts (Bélanger et al., 2019; Milla et al., 2022), demonstrating how individual motivations, ideological narratives, and social support systems interact to drive extreme behaviors. For instance, González et al. (2022) found that indicators of each of the three factors—Needs, Narrative, and Network—were present in individuals convicted of terror offenses. Ellenberg et al. (2023) applied the 3N framework to a sample of self-identified incels, identifying three subtypes of incels: hopers, internalizes, and externalizers. Externalizers, characterized by strong support for violence, were particularly influenced by their lack of a supportive network (social isolation) and their admiration for incel perpetrators. These findings highlight the utility of the 3N model in explaining incels’ harmful attitudes and behaviors.

Building on this work, we adapt the 3N framework to examine how poor mental health, incel ideology, and engagement with incel forums predict harmful attitudes and beliefs. While not a direct test of the 3N framework, consideration of these three core factors offers a useful lens for understanding the factors driving harmful attitudes among incels. In addition, our theoretical framework also examines antecedent factors that may lie upstream of our 3N-inspired factors. These factors include dispositional traits (e.g., personality) and experience (e.g., learned mate value) that might lead one to adhere to incel ideology, network with other incels, and develop poor mental health. On an exploratory basis, we included some such antecedents in the model, focusing on traits and experiences previously found to be higher among incels in extant research. For example, traits like low self-perceived mate value (Costello et al., 2023) and early life experiences such as bullying during adolescence are commonly reported among incels (Moskalenko et al., 2022).

We hypothesize that these antecedents contribute to harmful attitudes and beliefs through pathways that influence adherence to incel ideology, social engagement within incel communities, and poor mental health. These factors align with findings in radicalization research suggesting that personal grievances, humiliation, and social exclusion often precede engagement with extremist ideologies (Webber & Kruglanski, 2017). In the current research, we quantify harmful attitudes and beliefs using a composite measure of displaced aggression, misogyny (e.g., hostile sexism), and justification of violence. These measures not only constitute harmful attitudes in their own right, but have also been linked to real-world harm, such as sexual and domestic violence (O’Connor, 2023; Ruddle et al., 2017).

In sum, we investigate how incels’ poor mental health, ideological adherence, and networking predict these harmful attitudes, while also exploring the antecedent factors that may predispose incels to harmful attitudes and beliefs. While previous research on incels has been largely descriptive, this study advances the field by proposing a model of incel harm that incorporates antecedent factors, with the possibility of identifying specific pathways to harm.

### Interconnections Between Poor Mental Health, Networking, and Ideological Adherence

Our framework posits that mental health, ideology, and networking are not isolated factors but interrelated and mutually reinforcing. For example, individuals with compromised mental health (e.g., depression, anxiety) may gravitate toward incel forums to combat loneliness (Sparks et al., 2024a, 2024b). These forums offer validation through a shared narrative that frames their grievances as a result of injustice, reinforcing their victimhood lens (Costello et al., 2024b; Rousis et al., 2023). Such interactions may amplify extreme views, degrade mental health, and foster harmful attitudes (Ribeiro et al., 2021).

Ellenberg et al. (2023) observed this dynamic among externalizers, a subgroup of incels with the highest support for violence, who reported feeling more violent after participating in incel forums. These findings align with radicalization research suggesting that social exclusion and humiliation often precede engagement with extremist ideologies (Webber & Kruglanski, 2017). Our study builds on these insights, hypothesizing positive associations between poor mental health, networking, and ideological adherence in predicting harmful attitudes.

**Fig. 1:**
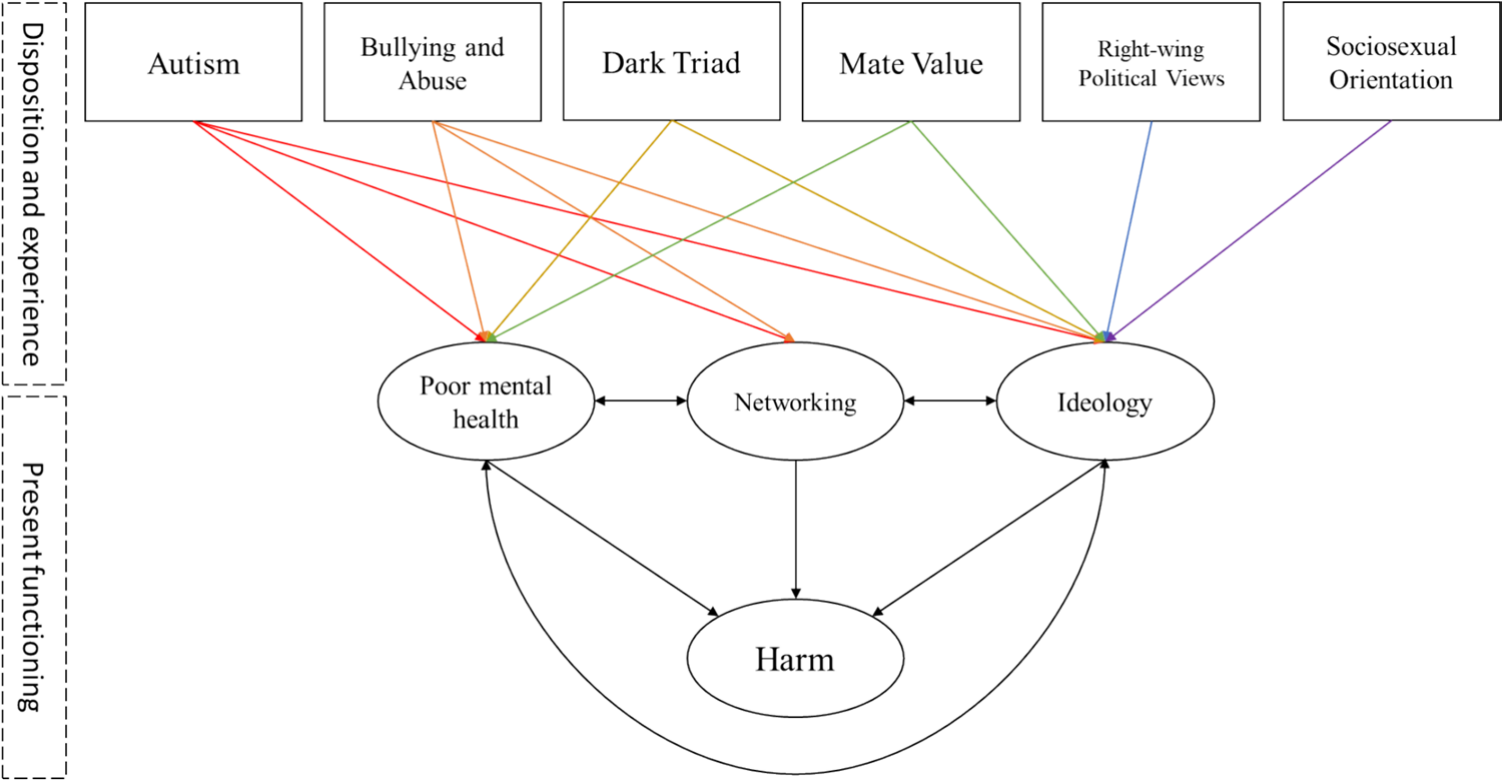
Predicting incel harmful attitudes and beliefs based on their poor mental health, usage of incel social networks, and adherence to ideology. Dispositional and experiential factors are included as an additional layer, predicting harm indirectly via the three predictors

These interconnections suggest that all combinations of the three factors should be positively associated. For example, stronger adherence to incel ideology is likely to correlate with higher levels of networking within incel communities. Similarly, higher levels of poor mental health (e.g., depression, anxiety) are expected to correlate with both greater ideological adherence and networking behaviors (see Fig. 1).

#### *Prediction 1*

Poor mental health, networking, and ideological adherence will each predict harmful attitudes and beliefs, and all combinations of the three factors will show positive covariance

### Pathways to Harm: Dispositional and Experiential Factors

In addition to considering the positive associations between factors of incel poor mental health, networking, and ideology, we now consider some of the dispositional and experiential traits that may predict harm indirectly.

### Political Beliefs

Although incels self-report being slightly left-leaning on average (Costello et al., 2022), those leaning right could be susceptible to incel ideology due to its anti-feminist narrative (Ging, 2019). Incels with right-wing leanings may be more predisposed to harmful attitudes and beliefs than those leaning left, as right-wing radical acts are generally more violent (Jasko et al., 2022).

### Dark Triad and Sociosexuality

The Dark Triad (psychopathy, narcissism, and Machiavellianism), although understudied in incels, is associated with misogyny (Douglass et al., 2023) and violence (Pailing et al., 2014). Such traits might attract individuals toward incel ideology.

Incels are characterized by a mismatch between their sociosexual desire (i.e., their desire for uncommitted sexual relationships) and their sociosexual behavior (i.e., their ability to engage in such relationships (Costello et al., 2022). This discrepancy may contribute to the development of resentful attitudes and harmful beliefs, as unmet sexual desires are linked to feelings of frustration and hostility (Costello et al., 2023). High levels of sociosexuality, often conceptualized as a desire for "impersonal sex," have been associated with a range of problematic mating-related attitudes, beliefs, and behaviors which cause harm to others. These include sexual harassment, errors in cross-sex mind reading, and voyeuristic and exhibitionist tendencies (Thomas et al., 2021). Recent research by Freyth and Jonason (2023) further underscores the connection between sociosexuality and harmful behaviors, demonstrating that Machiavellianism and psychopathy are strongly linked to risky sexual behaviors (e.g., lack of condom use) and the desire for casual sexual encounters. Moreover, sociosexuality is a key variable in theoretical models of sexual aggression and harm, such as the confluence model (Malamuth, 1996), which posits that high levels of sociosexuality, coupled with other risk factors, such as hostile sexism, predict sexual aggression.

Given previous research demonstrating higher sociosexual desire among incels (Costello et al., 2022), we included sociosexuality as an exploratory variable in this study to shed light on how this mismatch might fuel negative attitudes and beliefs, as well as behaviors that contribute to harmful outcomes.

### Autism Spectrum Disorder, Bullying and Abuse, and Low Mate Value

The prevalence of autism among incels (~ 18%; Speckhard & Ellenberg, 2022) significantly surpasses the general population's 1% base-rate (Zeidan et al., 2022). Autistic traits, such as difficulty empathizing (Baron-Cohen, 1990), and black-and-white thinking when faced with the uncertainty that is typical of romantic contexts (Suzuki & Hirai, 2023), may contribute to incel identification. Other features of autism, including challenges in forming attachments and high stress levels, could exacerbate susceptibility to incel ideology (Rousseau et al., 2023). Moreover, individuals with autism may be drawn to extremist groups due to a sense of belonging and admiration for their specialized knowledge (Lazzari et al., 2019; Walter et al., 2021). Incel ideology may also be particularly appealing to some individuals with autism because they offer clear “scripts” or “rules to live by” (Al-Attar, 2020; Krasenberg & Wouterse, 2019). For instance, the incel ideology offers a simplified explanation as to why they are unable to attract a partner. There is also some evidence that there is a “pipeline” whereby the same users of pick-up-artist forums eventually migrate to incel forums (Ribeiro et al., 2021). Young men, particularly men with autism, who try and fail to navigate a complex and dynamic dating market with the crude tactics learned in pick-up-artist forums, may reach the conclusion that they are incels.

A history of being bullied may also influence incel ideology adoption. Roughly 86% of incels say they have experienced some bullying (Moskalenko et al., 2022) compared to 33% of the general population (Lereya et al., 2015). Incels who have experienced bullying might harbor strong victimhood perceptions, leading them to feel entitled to harm others (Costello et al., 2022). Rousseau et al. (2023) found a higher prevalence of incel ideology among individuals with autism (particularly those who have experienced bullying) compared to neurotypical individuals (58.3% vs. 12.9%, respectively). Additionally, incels with autism and a history of bullying were more likely to idolize incel perpetrators and fantasize about violence (Moskalenko et al., 2022).

Finally, the misogynistic incel ideology likely reflects individual incels’ low sense of self-perceived mate value (Costello et al., 2023), as men who doubt their appeal to women or experience unwanted celibacy often endorse hostile sexism (Bosson et al., 2022; Grunau et al., 2022).

#### *Prediction 2*

All dispositional and experiential factors will predict harm indirectly via ideological adherence

Autism's comorbidity with mental health issues is well-documented (Hollocks et al., 2019). Meanwhile, an evolutionary perspective suggests failing in fundamental goals like mating can detrimentally impact mental health, with self-perceived mate value serving as a proxy for self-esteem in men (Schmitt & Jonason, 2019). Incels' mental wellbeing is adversely affected by their poor mating performance (Apostolou et al., 2024), possibly leading to misogynistic views (Grunau et al., 2022).

Although there is a relationship between the dark triad and depressive symptoms (Gómez-Leal et al., 2019), we know of no theoretically grounded reason to expect right-wing political views to predict poorer mental health outcomes. Similarly, a more unrestricted sociosexuality does not appear to directly affect mental health, although a discrepancy between sociosexual-desire and behavior often results in low wellbeing and resentment (Michalos, 1985).

#### *Prediction 3*

Autistic traits, bullying and abuse, dark triad, and low mate value will predict harm indirectly via poor mental health

Those with autism may also prefer online networking to in-person social interaction due to there being fewer complex social cues to navigate (Williams et al., 2021). The incel forums can also offer the individual with autism a sense of community, belonging, social significance and peer support that they do not experience elsewhere (Rousseau et al., 2023). Troublingly, individuals who reported stronger autistic traits were more likely to report that participating in incel forums made them feel violent and misogynistic (Speckhard & Ellenberg, 2022).

Incels (who may have experienced bullying) have a need for recognition (Costello et al., 2022) and prefer verifying their victimhood with peers (Rousis et al., 2023). Networking in incel forums could intensify harmful attitudes, as roughly 82% of incel forum threads are considered misogynistic (Halpin et al., 2022).

#### *Prediction 4*

Autistic traits and bullying experiences will influence harmful attitudes and beliefs indirectly via incels’ social networking

## Method

### Participants

After extensive data screening (see below), we recruited a total of 561 male participants. Of these, 64.5% were from the USA and the remainer from the UK. As can be seen in Table 1, on average, incels were in their mid-20s (*M*_age_ = 25.92 years, *SD* = 6.6), heterosexual, and childless. Most of the sample was white, but 42% self-identified as a person of color. The vast majority (85.6%) of participants selected “Involuntarily celibate” as their reason for singlehood, though a minority (12.7%) selected “By choice.”^1^ Typically, they were from a middle or lower-middle class background, had some form of post-secondary education, and were either living at home or renting. Most incels were either in full-time employment (42.4%) or education (16.4%). Compared to American incels, British incels in the sample were slightly older, more diverse in sexuality, from lower socioeconomic backgrounds, less ethnically diverse, better educated, less likely to be living at home, and more likely to select “by choice” as the man reason for their singlehood. However, all of these differences were small in size. As a group, participants were slightly left leaning in their political orientation (Table 2).

**Table 1:** Demographic characteristics for the UK and US subsamples

Characteristic	Total sample (*N* = 561)	UK (*n* = 199)	US (*n* = 362)	*t*/*χ*^2^ (df)	*p*	*d/V*
Age, mean (*SD*)	25.92 (6.60)	26.86 (6.46)	25.41 (6.62)	2.52 (559)	.01	0.22
Height (cm)	174.10 (10.78)	174.46 (11.50)	173.9 (10.38)	0.59 (555)	.55	0.05
*Sexual orientation*				10.41 (3)	.02	.14
Heterosexual	520 (92.7%)	181 (91%)	339 (93.6%)			
Homosexual	10 (1.8%)	7 (3.5%)	3 (0.8%)			
Bisexual	24 (4.3%)	6 (3%)	18 (5%)			
Other	7 (1.2%)	5 (2.5%)	2 (0.6%)			
*Socio-economic status*				9.48 (4)	.05	.13
Upper	12 (2.1%)	1 (0.5%)	11 (3%)			
Upper-middle	82 (14.6%)	21 (10.6%)	61 (16.9%)			
Middle	228 (40.6%)	82 (41.2%)	146 (40.3%)			
Lower-middle	152 (27.1%)	62 (31.2%)	90 (24.9%)			
Lower	87 (15.5%)	33 (16.6%)	54 (14.9%)			
*Fatherhood*				11.19 (2)	< .01	.14
No children	549 (98%)	189 (95.5%)	360 (98%)			
1 child	8 (1.4%)	6 (3%)	2 (0.6%)			
2 children	3 (0.5%)	3 (1.5%)	0 (0%)			
*Ethnicity*				12.84 (5)	.03	.15
White	326 (58.1%)	128 (64.3%)	198 (54.7%)			
Black	88 (15.7%)	32 (16.1%)	56 (15.5%)			
Mixed Race	34 (6.1%)	12 (6.0%)	22 (6.1%)			
South Asian	38 (6.8%)	11 (5.5%)	27 (7.5%)			
Hispanic	21 (3.7%)	1 (0.5%)	20 (5.5%)			
Other	54 (9.6%)	15 (7.5%)	39 (10.8%)			
*Education*				21.93 (4)	< .001	.20
Did not graduate high school	44 (7.8%)	22 (11.1%)	22 (6.1%)			
Graduated high school	133 (23.7%)	33 (16.6%)	100 (27.6%)			
Completed some FE/HE	191 (34%)	58 (29.1%)	133 (36.7%)			
Completed undergraduate degree	142 (25.3%)	59 (29.6%)	83 (22.9%)			
Completed postgraduate degree	51 (9.1%)	27 (13.6%)	24 (6.6%)			
*Living arrangements*				12.44 (4)	.01	.15
Homeowner	44 (7.8%)	23 (11.6%)	21 (5.8%)			
Renting alone	142 (25.3%)	50 (25.1%)	92 (25.4%)			
Renting with others	93 (16.6%)	40 (20.1%)	53 (14.6%)			
Living with parents/grandparents	278 (49.6%)	86 (43.2%)	192 (53%)			
Other	4 (0.7%)	0 (0%)	4 (1.1%)			
*Singlehood reason*				25.25 (2)	< .001	.21
By choice	71 (12.7%)	44 (22.1%)	27 (7.5%)			
Involuntarily celibate	480 (85.6%)	151 (75.9%)	329 (90.9%)			
Temporary (between relationships)	10 (1.8%)	4 (2%)	6 (1.7%)			
*Not in Employment, Education, or Training*				8.28 (5)	.14	.12
Full-time employment	238 (42.4%)	87 (43.7%)	151 (41.7%)			
Full-time education	92 (16.4%)	28 (14.1%)	64 (17.7%)			
Part-time employment	71 (12.7%)	32 (16.1%)	39 (10.8%)			
Part-time education	27 (4.8%)	7 (3.5%)	20 (5.5%)			
Employment-education mix	33 (5.9%)	7 (3.5%)	26 (7.2%)			
None	100 (17.8%)	38 (19.1%)	62 (17.1%)

**Table 2:** Percentage of sample which sided with a right-wing belief over a left-wing alternative

Political belief (abridged)	Total sample (*N* = 561)
1. Government wasteful/inefficient	341 (60.8%)
2. Government regulation of business harmful	210 (37.4%)
3. Poor people have it easy because of benefits	224 (39.9%)
4. Government can't do more for needy	283 (50.4%)
5. Ethnic minorities responsible for their outcomes	343 (61.1%)
6. Immigrants today are a burden	256 (45.6%)
7. Military strength brings peace	228 (40.6%)
8. Corporations profits are fair	208 (37.1%)
9. Environmental regulations hurt economy	243 (43.3%)
10. Homosexuality should be discouraged	227 (40.5%)
Mean score (*SD*)	1.54 (0.25)

*Note* The mean score ranges from 1 (Extremely right wing) to 2 (Extremely left wing). A score of 1.5 indicates a balance between right-wing and left-wing responses. The sample average of 1.54 is significantly left of center, but only slightly so, *t*(560) = 4.03, *p* < .001, *d* = 0.17.

Described as a study of “The impact of social networking on incel attitudes, beliefs, and mental health,” incels were recruited using a mixture of social media, podcast promotion, and via incel forums. Incels are considered an extremely hard to reach group who are cynical of the motives of academic researchers. Other research teams have had to collaborate with the administrator of the largest incel forum; incels.is, even including them as a coauthor (see Speckhard et al., 2021). This is a recruitment strategy that has raised ethical concerns and doubts about collusion (Carian et al., 2023). Our research team maintained a collegial rapport with the administrator of incels.is, but they did not have any input into the study design, nor were they made aware of any of our hypotheses. Nevertheless, they agreed to circulate a link to our survey to the forum users by direct message and forum posts.

Potential participants who clicked on the link were sent to a single-page website containing a research mission statement, biographies of researchers, and examples of their existing publications and media interviews. The web page contained a link to a screening questionnaire that provided the full study link to participants who were (1) over 18, (2) an incel, (3) a UK/US resident, and (4) aware of the sensitive and potentially distressing content of the questionnaire.

Participants who completed the study were offered either £20 (UK) or $20 (US) compensation. To keep the data anonymous, payment was handled by a third-party payment company. Once they completed the study, participants were given a random code to provide to the payment company along with their personal details and bank account information for payment. Each week, the submitted completion codes were sent to the research team for verification before processing payment. Thus, the researchers had no access to the participant’s personal data, and the payment company had no access to survey responses. Because some incels might have been uncomfortable providing their personal data, we gave them the option to donate their compensation to a men’s mental health charity (Movember) instead. A total of 125 (22.5%) did so.

### Procedure

Participants were presented with an information sheet and consent form, explaining how their data would be handled, and registering their informed consent. Next, they were taken to a screen explaining the payment process and how it protected their anonymity. They then completed a demographic questionnaire which also contained a political orientation measure adapted from the Pew Research Centre (2014). This measure gave participants 10 pairs of statements. For each pair, they had to pick the option which best matched their views. Each pair was about a different political issue (e.g., immigration, government regulation of business, supporting the poor) and one statement gave a right-leaning view (e.g., “Government regulation of business usually does more harm than good”) while the other gave a left-leaning view (e.g., “Government regulation of business is necessary to protect the public interest”). We changed “Black people” to “Ethnic Minorities” in the question about racial discrimination to make this more applicable to both US and UK participants.

#### Dispositional and Experiential Antecedents

We gave participants several measures to record their psychological disposition, and experience. In addition to political orientation (see above), they completed the AQ-10 (Allison et al., 2012) which is a tool used by primary care providers in the UK to guide autism referrals. The personality traits of sociosexuality and dark triad were recorded using the Sociosexual Orientation Inventory-Revised (Penke & Asendorpf, 2008) and The Dirty Dozen (Jonason & Webster, 2010) respectively, while self-perceived mate value was recorded using the Mate Value Scale (Edlund & Sagarin, 2014). Finally, experience of bullying and abuse was measured using the items “How often did you experience bullying as a child?” and “How often did you experience abuse from adults when you were a child”, both answered using a 1 (*None at all*) to 5 (*A great deal*) scale.

#### Operationalization of Harm

In this study, harm was conceptualized along two primary dimensions relevant to healthcare providers, anti-extremism agencies, and the general public. The first dimension involved traits associated with a risk of physical acts of violence. Due to ethical and methodological constraints, we could not measure a direct propensity for violent acts. Instead, we assessed traits indicative of such risk, such as condoning violence in the name of their cause and tendencies toward angry rumination about perceived harm inflicted on incels. The second dimension pertained to the perpetuation of harmful misogynistic attitudes and beliefs, which we captured using measures of hostile sexism and rape myth acceptance. These attitudes, while not exclusive to the incel community nor uniformly held among its members, are highly relevant to understanding incel-related harm and were therefore included as dependent variables in our analysis. To measure misogynistic views, we used the Hostile Sexism questions from The Ambivalent Sexism Inventory (Glick & Fiske, 2018) and the short Illinois Rape Myth Acceptance (IRMA) Scale (Bendixen & Kennair, 2017). Displaced aggression was measured using the Displaced Aggression Questionnaire (Denson et al., 2006).

#### Mental Health

To measure mental health, we used the PHQ-9 (Kroenke et al., 2001) for depression and GAD-7 (Spitzer et al., 2006) for anxiety, which are tools commonly used by clinicians to screen for these mental health issues. For the former, a score of 15 or more indicates moderately severe depression and for the latter, a score of 10 or more indicates moderate anxiety. These are both the second highest level of each scale and suggest clinically significant symptoms. Loneliness and sensitivity to rejection were measures using a three-item scale of loneliness (Hughes et al., 2004) and the eight-item version of the Rejection Sensitivity Questionnaire (Downey & Feldman, 1996).

#### Incel Ideology

The next part of the questionnaire recorded adherence to incel ideology using custom-made items. Participants were first asked whether they considered themselves part of the incel community and, if so, for how long they had been part of it. They were then asked whether they felt there was such as a thing as “incel ideology” as a “Yes” or “No” question. They were also asked to rate, using a 1 (*Strongly disagree*) to 5 (*Strongly agree*) scale, to what extent they felt incels had a shared worldview. Next, they were presented with a description of a popular incel belief:

Some incels believe in biological determinism, more specifically in the 80/20 rule, which states that 80% of women desire only 20% of men. In this view, men who are usually good looking, muscular, tall, and wealthy, or have otherwise high status (e.g., "Chads") are popular among attractive, sexually promiscuous women who are vain and obsessed with jewelry, makeup, and clothes (e.g., "Stacys"). Men who do not fit the description of a Chad are destined for a life of loneliness and will never have a willing sexual partner or a meaningful intimate relationship.

Participants were asked to what extent they agreed with this statement using a 1 (*Strongly disagree*) to 5 (*Strongly agree*) scale and whether they thought most incels agreed with it using “Yes” or “No” responses. Next, they were presented with seven groups in a random order (Women; Non-incel men; Wider society; The political right; The Political left; Feminists; and Incels themselves) and asked to what extent they thought each was an enemy of the incel community. They responded using the same 1 (*Strongly disagree*) to 5 (*Strongly agree*) scale. The next question asked incels whether they felt violence against those who caused harm to incels was ever justified:

Some people think that violence against other people is justified if they are causing harm to incels. Other people believe that no matter what the reason, this kind of violence is never justified. Do you personally feel that this kind of violence is often justified, sometimes justified, rarely justified, or never justified?

Responses were given using a Never (1), Rarely (2), Sometimes (3), and Often (4) scale. Finally, participants were asked nine questions aimed at measuring their perceptions of incels as a discriminated against group. This measure was adapted from Wolfowicz et al. (2023) who used a similarly worded item about Islam when studying jihadist extremism.

#### Incel Networking

The final part of the questionnaire addressed the interactions between incels on social networks. Participants were shown seven different networking methods in a random order: Anonymous forums/social media (e.g., 4chan); Regular forums/social media (with accounts); in person; Telephone calls; Video conferencing (e.g., Zoom, Teams); Discord; and Messaging services (e.g., WhatsApp, Telegram). Then, they were asked to think about their contact with other incels over the last year and assign a percentage of their total contact time to each. Follow-up questions were asked about any platform given a percentage greater than 0. These follow-up questions pertained to experiences over a typical week, and asked participants how many hours they spent using the platform and how many incels they interacted with using it. These two frequency questions were recorded using a pseudo-exponential scale from 1 (less than an hour, 0 people) to 9 (more than 33 h, more than 20 people). All other items were recorded on a five-point Likert scale, with higher numbers including greater agreement or higher frequency. These items related to how often the participant came across radical people and radical content and, for the anonymous and pseudonymous forums/social media platforms only, how often they engaged with other users and created content. Finally, for each method they were asked six items related to feelings of acceptance and social support felt when using the methods, using a 1 (*Strongly disagree*) to 5 (*Strongly agree*) scale, which were averaged to produce one number representing feelings of social support for that medium.

Before exiting the questionnaire, the participants were given a reminder of the payment process and given the option to donate to charity if they wished. After making their choice, participants were taken to a debrief form which contained their unique completion code and link to the payment provider website. We report all manipulations, measures, and exclusions in these studies.

### Data Quality and Sample Size

Our intention was to recruit 250 incels from the UK and 250 from the USA to provide stable effect size estimates for each subgroup (Schönbrodt & Perugini, 2013). The data were subject to rigorous screening practices to identify duplicate responses and responses submitted without due care and attention or from those who did not meet the inclusion criteria (e.g., participants who said they were in committed relationships, not from the USA or UK, etc.). Because participants who completed the survey and requested payment had to provide personal and bank account details to payment company, the company was able to make us aware of the ID numbers of duplicate submissions while retaining anonymity. When duplicate submission were identified, the first response was retained in the dataset and all others removed. We also included some variables in the demographic form (such as relationships status and country) that allowed us to detect spam responses (e.g., those who said they were female, married, and from Algeria, despite earlier indicating they were male, single, and from either the UK or USA). Together, we detected and removed 103 submissions (15.5% of the responses). In addition to removing noise from the data, this raises questions about the potential for bias in other incel research with no verification protocol.

### Preregistration

Due to its sensitive nature, we did not preregister this work. However, in advance of this research, we created a research project website containing a mission statement, links to previous research, media interviews, information about our funding, etc. This was done in an effort to be as transparent as possible with our target population of participants, who are often skeptical of academic research. As made clear in our mission statement on our research project website, the data we gather is not shared with third parties. We took this stance on data sharing to alleviate the concerns of the incel community who are incredibly skeptical of government bodies and researchers. Indeed, we believe that making this commitment, and therefore becoming ethically obliged to not share data with our funder or the wider public, is one of the reasons why we were able to gather such a large sample.

## Results

### Dispositional Traits and Experiences

Descriptives for these traits, some previously unmeasured in incels, can be seen in Table 3. Approximately 30% had an AQ-10 score that would meet the clinical referral threshold for an autism assessment. As a group, incels had similar dark triad scores as large samples of undergraduates (e.g., Jonason & Webster, 2010). However, they scored lower on sociosexuality (Penke, 2011) and mate value (Edlund & Sagarin, 2014). Finally, a significant minority of incels said they had experienced relatively high levels of bullying (13.3%) and abuse by adults (5.9%) in childhood.

**Table 3:** Incel mental health, disposition, and experience

Characteristic	Total sample (*N* = 561)
*Mental health*	
PHQ-9^a^, M (*SD*)	13.03 (6.93)
PHQ-9, % over 15 points	218 (38.9%)
Suicidal thoughts, % nearly every day	121 (21.6%)
GAD-7^b^, M (*SD*)	8.99 (5.81)
GAD-7, % over 10 points	241 (43%)
Loneliness^c^, M (*SD*)	2.54 (0.56)
Rejection sensitivity^d^, M (*SD*)	13.80 (5.72)
*Disposition and experience*	
AQ-10^e^, M (*SD*)	4.46 (2.28)
AQ-10, % over 6-points	172 (30.7%)
SOI-R^f^, M (*SD*)	3.66 (1.36)
Dirty Dozen^g^, M (*SD*)	3.11 (1.08)
Mate Value^h^, M (*SD*)	2.67 (1.22)
Bullying^g^, M (*SD*)	2.8 (1.24)
Bullying, % “A great deal”	74 (13.3%)
Abuse^g^, M (*SD*)	2.18 (1.18)
Abuse, % “A great deal”	33 (5.9%)

*Note:* Minimum and maximum scores for each measure: ^a^0–27, ^b^0–21, ^c^1–3, ^d^1–36, ^e^0–10, ^f^1–9, ^g^1–5, ^h^1–7

### Harmful Attitudes and Beliefs

Compared to population estimates for young men (Bendixen & Kennair, 2017), incels scored higher on both hostile sexism and rape myth acceptance, two traditional measures of misogyny (Table 4). In terms of their relationship with anger, incels had higher levels of angry rumination than population samples as well as revenge planning. However, they had lower levels of displaced aggression compared to a similar age group (Denson et al., 2006). Finally, a substantial proportion of incels felt that violence was “Sometimes” (20%) or “Often” (5%) justified against those who sought to harm them.

**Table 4:** Incel ideology and harm variables

Characteristic	Total sample (*N* = 561)
*Ideology*	
Feelings of discrimination, M (*SD*)^a^	3.45 (1.00)
Believes incel ideology exists, % agree	383 (68.3%)
Incels share the same worldview, % agree	341 (60.8%)
Personal agreement with 80/20 view, % agree	360 (64.2%)
Incels agree with 80/20 view, % agree	454 (80.9%)
Enemy—Feminists, M (*SD*)^a^	4.35 (0.98)
Enemy—The political left, M (*SD*)^a^	3.93 (1.10)
Enemy—Wider society, M (*SD*)^a^	3.85 (1.12)
Enemy—Women, M (*SD*)^a^	3.75 (1.19)
Enemy—Non-incel men, M (*SD*)^a^	3.19 (1.20)
Enemy—Incels themselves, M (*SD*)^a^	3.06 (1.38)
Enemy—The political right, M (*SD*)^a^	3.04 (1.15)
Identify a part of the Incel community, %	411 (74%)
Months in community, median (IQR)	41 (44.75)
Incel longer than 3 years or more, %	251 (44.7%)
*Harm*	
Justification of Violence, M (*SD*)^b^	1.82 (0.93)
Justification of Violence, % sometimes and often	138 (24.6%)
Justification of Violence, % often	31 (5.5%)
Angry Rumination^c^	4.21 (1.66)
Revenge Planning^c^	3.41 (1.55)
Displaced Aggression^c^	2.39 (1.18)
Hostile Sexism^a^	3.17 (1.03)
Rape Myth Acceptance^a^	2.81 (1.20)

*Note* Minimum and maximum and minimum scores for each measure: ^a^1–5, ^b^1–4, ^c^1–7

### Mental Health

Consistent with previous research (e.g., Costello et al., 2022), incel mental health was poor (Table 3). More than a third of the sample met the criteria for moderate depression or anxiety. Levels of loneliness were also high; 48% of participants selected the highest response for all three items. Incels were also particularly sensitive to rejection in this sample compared to the wider population (Downey & Feldman, 1996).

### Incel Ideology

There was a fair degree of ideological consistency within the sample (Table 4). Just over two-thirds of respondents agreed that incel ideology exists, and a similar proportion agreed that incels shared the same worldview. When asked about belief in the “80/20” principle (that 80% of women are only sexually interested in the most attractive 20% of men) almost two-thirds of incels agreed with it and four fifths felt that most incels agreed with it. Incels also typically believed that they were discriminated against, though the average response straddled “neither agree not disagree” and “somewhat agree.” Incels considered “Feminists” to be the biggest enemy of the community, followed by “The political left,” “Wider society,” and “Women.”

### Social Networking

As can be seen at the bottom of Table 5, most incels considered themselves part of the incel community and those who did had been a part of it for 41 months (median). Of the whole sample, almost half had identified as incel for three years or longer.

**Table 5:** Incel use of different social networks

Characteristic	Anonymous social media	Registered social media	In Person	Telephone	Video calls	Discord	Messaging apps	Average
*Within the last year*^a^								
Ever used	308 (54.9%)	286 (51%)	99 (17.6%)	37 (6.6%)	36 (6.4%)	172 (30.7%)	99 (17.6%)	1.85 (1.59)
Often used (over 25%)	229 (40.8%)	191 (34%)	35 (6.2%)	6 (1.1%)	7 (1.2%)	88 (15.7%)	35 (6.2%)	–
Primary (over 50%)	182 (32.4%)	149 (26.6%)	27 (4.8%)	1 (0.2%)	6 (1.1%)	54 (9.6%)	20 (3.6%)	–
*Within the last 2 weeks*								
Time spent	3.95 (2.03)	4.68 (1.99)	2.30 (1.76)	2.16 (1.19)	2.36 (1.31)	3.08 (2.05)	2.83 (1.81)	3.84 (1.67)
People engaged	5.79 (2.69)	5.16 (2.70)	2.66 (1.86)	2.51 (1.26)	3.44 (2.37)	4.48 (2.48)	3.55 (2.4)	4.85 (2.39)
Interaction	2.42 (1.09)	2.50 (1.08)	–	–	–	–	–	–
Content generation	1.88 (0.91)	1.84 (0.96)	–	–	–	–	–	–
Radical people	3.13 (1.10)	2.71 (1.11)	2.00 (1.13)	1.97 (0.96)	2.17 (1.13)	2.48 (1.26)	2.24 (1.19)	2.74 (1.01)
Radical content	3.10 (1.15)	2.7 (1.10)	1.95 (1.07)	1.97 (0.90)	2.11 (1.14)	2.49 (1.29)	2.27 (1.18)	2.71 (1.00)
Feelings of support	3.44 (0.92)	3.20 (1.05)	3.47 (0.93)	3.50 (0.93)	3.72 (0.81)	3.58 (0.92)	3.67 (0.75)	3.38 (0.83)

*Note* a = with the exception of the Average, these numbers reflect *n* (%). All other number reflect means (*SD*). Those who used a method within the last year were asked follow-up questions about their experience with the communication type within the last two weeks, including content creation and exposure to radical people and content

Incels used all the types of networking we asked about, including in person communication. Typically, they used two types of networks out of the seven we asked about and they were more likely to engage with methods allowing multi-person rather than one-to-one communication (e.g., video and telephone calls). The most common methods used within the last year were anonymous (e.g., 4chan) and registered (e.g., incels.co, Twitter) social media and forums, followed by Discord servers. These three methods also led to engagement with a greater number of incels, and greater exposure to radical people and content.

### Cultural Differences

This is the first study with subsamples of incels from two different countries in numbers large enough to allow for meaningful comparisons. There were some small differences in responses to specific political orientation items, loneliness, and perceiving incels as their own enemies. There were also some slightly larger (medium) differences in the types of social networks that were used, and self-reported exposure to extreme people and content. We do not dwell on these differences here, but overall, the samples were similar. See supplementary materials for more information (Table S1).

### Predicting Harmful Beliefs and Attitudes

#### Principal Components Analyses (PCA)

**Fig. 2:**
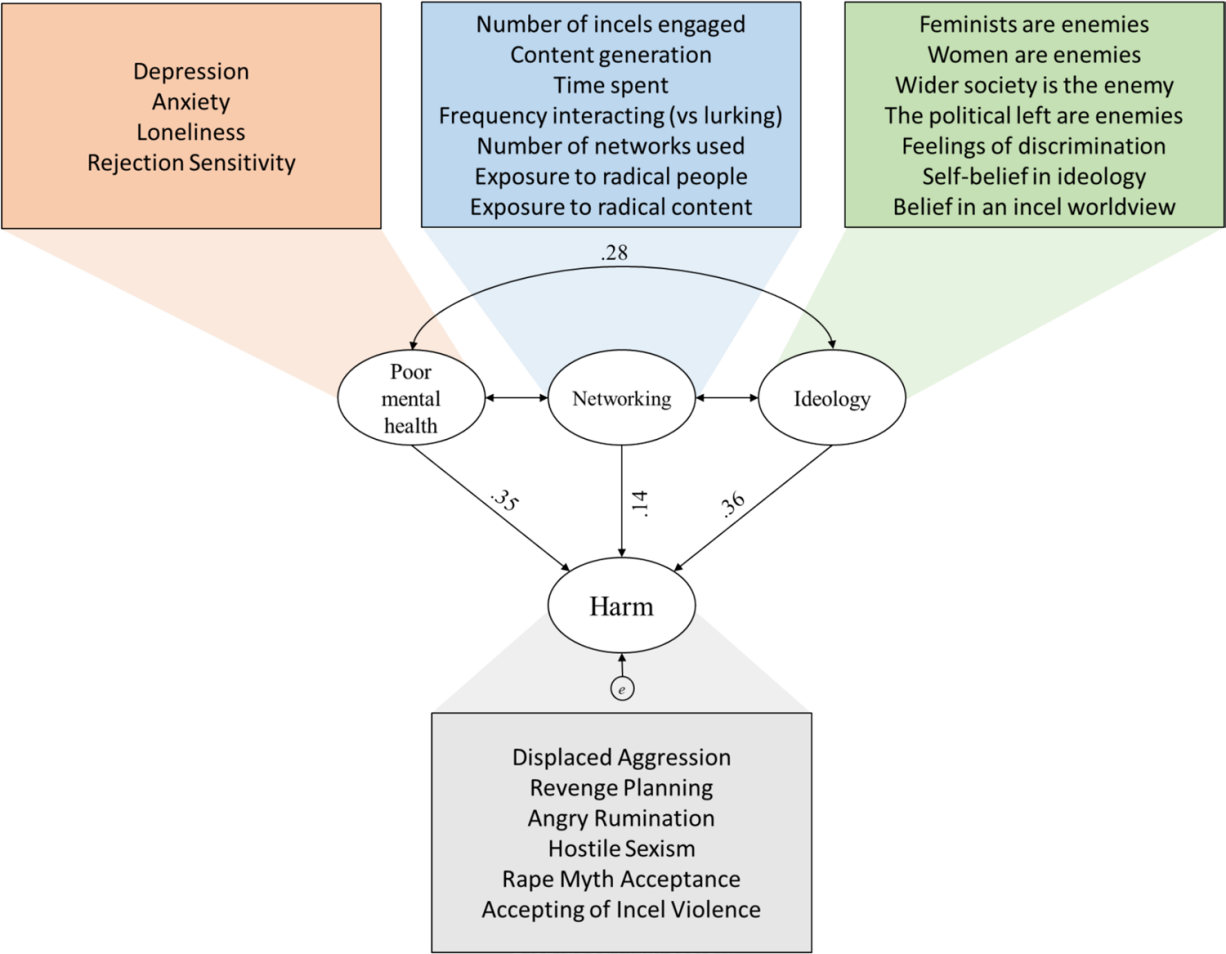
Outcome of a pathway analysis predicting harmful incel attitudes and beliefs using poor mental health, adherence to incel ideology, and quantity of incel social networking. All values represent standardized betas (all *p*s < .01)

In preparation for the pathway analysis, we created four variables using PCA (Fig. 2). First, “Harmful attitudes and beliefs” served as main dependant variable and was comprised of the three displaced aggression subscales, hostile sexism, rape myth acceptance, and the condoning of incel-defending violence. Second, “Poor Mental Health” was formed from the anxiety, depression, loneliness, and rejection sensitivity questionnaires. Third, “Ideological Adherence” was formed from the “enemy” ratings for the four top incel enemies (feminists, the political left, women, and wider society), self-reported belief in incel ideology, belief in a consistent incel worldview, and average responses to the incel discrimination scale. Finally, “Network Usage” was formed from the participant’s self-reported weekly network use summed across different platforms. The variables were total amount of time spent, how often interaction with other incels took place, how many incels were engaged with, and exposure to radical people/beliefs.

Each analysis was performed on the full sample, except for Network Usage which was conducted only on those who engaged in incel Networks within the last year (*n* = 377). Full results of the factor analyses can be found in Table S2. However, in all cases (1) KMO and Bartlett test results indicated that the data was appropriate for factor analysis, (2) Eigenvalues and scree plots pointed to a single factor solution, (3) more than 39% of the variance was accounted for by a single factor, and (4) all items loaded more than 0.38 on their respective factors. A standardized score was produced for each factor.

#### Preliminary Regression Analyses

Two regression models formed the groundwork for our larger pathway analysis. The first model predicted Harm using Poor Mental Health, Network Usage, and Ideological Adherence. This model accounted for 33% of the variance in Harm and all predictors were significant, though Network Usage showed the weakest effect. Adding sociosexuality, mate value, dark triad, combined childhood bullying and abuse (*r* = 0.51), political beliefs, and autism quotient significantly improved the model (*R*^2^ change = 0.25, *p* < 0.001). However, sociosexuality was not a significant predictor (Table 6).

**Table 6:** Regression analyses for poor mental health, network usage, and ideological adherence. Dispositional and developmental variables are included in the second model

Variable	Model 1				Model 2			
	*B*	*SE*	*t*	*p*	*B*	*SE*	*t*	*p*
Poor mental health	0.33	0.04	7.78	< .001	0.19	0.04	4.50	< .001
Network usage	0.14	0.04	3.29	< .01	0.11	0.03	3.24	< .01
Ideological adherence	0.38	0.05	7.97	< .001	0.27	0.04	6.84	< .001
Political orientation					− 0.67	0.14	− 4.65	< .001
Mate value					0.06	0.03	2.00	< .05
Sociosexuality					0.02	0.03	0.97	.33
Dark triad					0.38	0.03	11.10	< .001
Bullying and abuse					0.07	0.04	1.98	< .05
Autism quotient					0.06	0.02	4.12	< .001
Model	*F*(3, 374) = 62.03, *p* < .001, *R*^2^_adj_ = .33				*F*(9, 367) = 57.97, *p* < .001, *R*^2^_adj_ = .577			

#### Pathway Analysis

We first tested Prediction 1 using pathway analysis in AMOS for SPSS. As seen in Fig. 2, Mental Health, Network Usage, and Ideological Adherence predicted harm. Contrary to predictions, there was no covariance between Networking and Mental Health (Estimate = 0.019, *p* = 0.70) or Ideological Adherence (Estimate = 0.028, *p* = 0.60). With these removed the model remained an excellent fit to the data (*χ*^2^(12) = 0.34, *p* < 0.17; CFI = 1.00; RMSEA < 0.001).

**Fig. 3:**
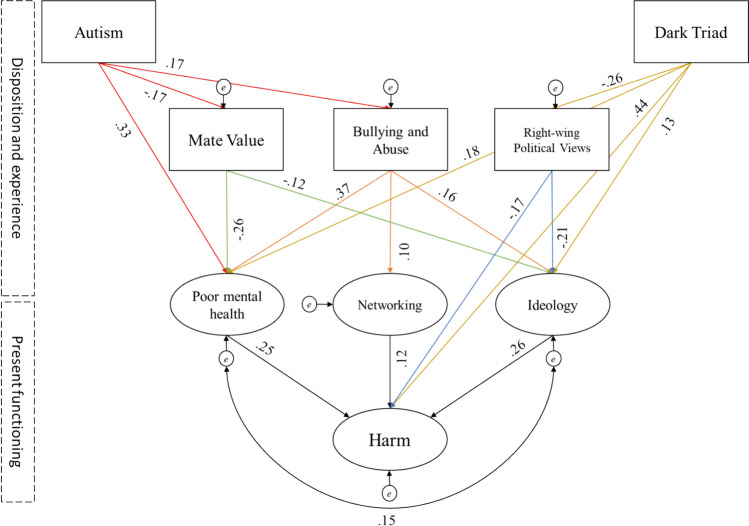
Outcome of the pathway analysis predicting harmful incel attitudes with the addition of dispositional and experience traits. All values represent standardized betas (all *p*s < .01)

Next, we added our experiential and dispositional variables as an additional layer, predicting Ideological Adherence, Networking, and Poor Mental Health in line with our predictions (Fig. 3). The resulting model was a poor fit (*χ*^2^(16) = 197.03, *p* < 0.001; CFI = 0.73; RMSEA = 0.17). Examining the modification indices revealed three ways of improving the model. First, by allowing autism to predicted by mate value and bullying. Second, by allowing dark triad to predict political beliefs. And finally, by allowing both dark triad and political beliefs to predict Harm directly. Adding these made the model a better fit (*χ*^2^(18) = 43.68, *p* = 0.001; CFI = 0.96; RMSEA = 0.06).

## Discussion

Concerns surrounding incels continue to grow (Blake & Brooks, 2023), with prevalent mental health issues (Costello et al., 2022) and several high-profile instances of incel-linked violence (Hoffman et al., 2020). Indeed, the numbers of men who are potentially vulnerable to incel ideology are growing. In a large cross-cultural database (*n* = 7181) from 14 different countries, 13% of participants identified as being involuntarily single (Apostolou et al., 2023). Given this pressing concern, our study, the largest of its kind to date, revealed that poor mental health, ideological adherence, and social networking significantly predict harmful attitudes and beliefs among incels, with poor mental health and ideology being twice as predictive as networking.

Building on our findings, we propose the dual pathways hypothesis of incel harm, which identifies two distinct trajectories—one rooted in dark triad personality and political beliefs, and another driven by autistic traits, poor self-perceived mate value, and experiential vulnerabilities (e.g., reported experiences of bullying and abuse)—that lead to harmful attitudes and beliefs. In the following sections, we discuss the implications of these findings for understanding incel-related harm, outline the practical applications of our findings for intervention strategies, and address the limitations of the study to inform future research.

### Incels’ Demographics

On average, incels were in their mid-twenties, heterosexual, and childless. Our sample was ethnically diverse, with 42% self-identifying as a person of color. Most participants considered themselves from a middle class or lower-middle class background. Most had some form of post-secondary school education. These demographics are consistent with other surveys of incels (e.g., Costello et al., 2022; Moskalenko et al., 2022).

### Incels’ Psychological Disposition, Poor Mental Health and Autism

Consistent with previous research (Costello et al., 2022), incel mental health was poor. More than a third of the sample met the criteria for moderate depression or anxiety (cf. Delaney et al., 2024). Levels of loneliness were also high; 48% of participants selected the highest response for all three loneliness items. Troublingly, there were daily thoughts of suicide in a fifth of the sample. These findings highlight a clear and urgent concern: the primary risk of harm among incels appears to be directed toward themselves. Indeed, two of the strongest correlates of male suicidal ideation are failure in heterosexual mating and burdensomeness to kin (de Catanzaro, 1995). Both factors are salient for incels, roughly 18% of whom report to be NEET (not in education, employment, or training) and 49.6% of whom report to still living with their parents into adulthood.

Incels' suicidal ideation is also concerning due to research suggesting that suicidal people often also harm others. Research indicates that approximately 5% of all homicidal deaths are attributed to murder-suicides, and in the USA, these incidents account for an estimated 1000–1500 violent deaths annually, translating to about 20–30 deaths each week (Marzuk et al., 1992). Although the vast majority (90–95%) of murder-suicide perpetrators are men (Logan et al., 2008), approximately 65–70% of these incidents involve intimate partners, often occurring amid a breakup or domestic conflict. Perhaps more immediately relevant to incels, data show that 71.2% of mass shooters were suicidal prior to, or intended to die during, the shooting (Peterson et al., 2021).

As a group, incels had similar dark triad (narcissism, psychopathy, and Machiavellianism) scores as large samples of undergraduate students (Jonason & Webster, 2010). However, they scored lower on sociosexuality (Penke, 2011), and self-perceived mate value (Edlund & Sagarin, 2014). Two measures of misogyny, hostile sexism (Glick & Fiske, 2018) and rape myth acceptance (Bendixen & Kennair, 2017) were also higher than population estimates. Finally, a significant minority of incels said they had experienced relatively high levels of bullying (13.3%) and abuse by adults (5.9%) in childhood.

In our study, incels exhibited elevated levels of angry rumination compared to general population samples. They also showed increased tendencies for revenge planning, albeit with slightly lower levels of displaced aggression compared to a similar age group (Denson et al., 2006). This suggests that while incels tend to dwell on seeking revenge against those they perceive as having wronged them, they appear less inclined to direct their frustrations toward those they see as innocent parties. Additionally, they demonstrated heightened rejection sensitivity, anticipating social rejection if they were to seek support (Downey & Feldman, 1996). These findings emphasize the importance of challenging incels belief that society hates them (Costello & Thomas, 2025; Daly & Reed, 2022).

Around a third of our sample meets the clinical cut-off for and autism referral. This extends our understanding beyond previous studies that relied on self-reported autism diagnoses (Speckhard & Ellenberg, 2022) and suggests that many incels have not yet received, but would qualify for, a formal assessment. Our pathway analysis showed that autism related traits were an indirect predictor of harmful attitudes and beliefs. Despite these findings, we echo Al-Attar's (2020) caution against assuming causality between autism and violence due to co-existing mental health issues, psychosocial adversities, and the heterogeneous nature of autism. It is important to highlight that not all men with autism are at risk of inceldom, but a particular subgroup of individuals in certain circumstances (perhaps those who have experienced bullying) may be more vulnerable (Broyd et al., 2023). Finally, we note that individuals with autism are not more likely to engage in violent crime when compared to the general population (e.g., Mouridsen, 2012). Future research should explore how autism might influence or be influenced by incel ideology and whether unique intervention approaches are required for this subgroup.

### Incels’ Ideological and Political Beliefs

There was general agreement among respondents that incel ideology exists; just over two-thirds of respondents believed this to be the case. The majority also agreed that incels shared the same worldview, though the proportion was slightly lower (61%). When asked about a specific belief (that 80% of women are only sexually interested in 20% of men) almost two-thirds of incels indicated that they personally agreed with it, while more than 80% felt that most incels agreed with it.

Some commentators have suggested a connection between incels and the extreme right wing (O’Malley & Helm, 2023), with ecosystem analysis indicating that the “manosphere” encompasses elements of the far right (Ribeiro et al., 2021). Using Pew Research’s “Ideological Consistency Scale,” this survey shows a complex picture. When asked about their political orientation through binary policy position questions, incels, on average, positioned slightly left of center. The only predominantly “right-wing” views shared by most incels were perceptions of governmental inefficiency and the belief that ethnic minorities are primarily responsible for their own circumstances. Conversely, they leaned substantially left of center on issues related to homosexuality, corporate profits, and social benefits. However, it is important to exercise caution before concluding that there is no overlap between the far right and incels. Concepts like “extreme right-wing” are complex and cannot be easily simplified into a set of policy questions. Our findings suggest that, concerning public policy matters at least, incels do not seem particularly right wing as a group. This aligns with earlier research indicating that around 63% of incels self-reported a left-leaning or centrist political affiliation (Costello et al., 2022).

A key part of ideology is the idea of clearly identified “out-groups” who are often held responsible for the “in-groups” woes, and in some cases, are considered a viable (or even mandatory) target of extremist violence (Berger, 2017; Ingram, 2016). Incels considered “Feminists” to be their community’s biggest enemy, followed by “The political left,” “Wider society,” and “Women.” The identification of ‘out-groups’ as primary enemies is consistent with the Narrative component of the 3N Framework, which posits that individuals adopt ideological grievances to justify their in-group's plight and externalize blame (Webber & Kruglanski, 2017). This process can make violence appear not only permissible but morally obligatory. Ellenberg et al. (2023) further illustrated this dynamic by identifying incels who strongly endorsed the belief that their grievances, particularly those associated with feminism and societal expectations, were the fault of these out-groups. These narratives provide a simplified lens through which complex societal and interpersonal failures are interpreted, fostering hostility and justifying harmful attitudes.

At first glance our findings about incels’ enemies may appear to conflict with the findings which suggest that incels are slightly left of center politically. However, it should be noted that the political spectrum questions did not relate specifically to the titles of “left” or “right”, but rather asked a series of policy questions. Therefore, terms such as these may carry positive or pejorative connotations without directly relating to policy preferences. Moreover, this sample was only slightly left of center, which opens the possibility that they conceptualized “the political left” as those who they see as considerably more left wing.

### Online and Offline Networking Among Incels

Most incels primarily communicate through anonymous online platforms, with only 18% engaging in any face-to-face interactions, and just 5% relying on in-person communication as their main mode. This contrasts with the assumption that incels solely inhabit online spaces, unlike other extremist-supporting groups (Whittaker, 2021). However, caution is needed in interpreting these findings, as there is no evidence of significant offline mobilization. It is possible that individuals reported in-person interactions due to acquaintances offline who also identify as incels. Future research could investigate the nature of these offline interactions, including whether they involve long-standing friendships. The median community membership duration was 41 months, with nearly half identifying as incels for over three years. Participants generally felt somewhat supported and accepted on social media, which may foster identity fusion (Rousis et al., 2023), potentially contributing to individual violence if endorsed by the group (Webber & Kruglanski, 2017).

Online networks play a critical role in radicalization by providing an environment where personal views and grievances are validated and amplified (Kruglanski et al., 2019). This aligns with findings from Ellenberg et al. (2023), who observed that incels with the highest support for violence (“externalizers”) reported lacking formative friendships and described online incel forums as a source of reinforcement for violent tendencies. This emphasizes how such networks serve as echo chambers, intertwining personal identity with collective ideology, thus deepening commitment to incel beliefs (Rousis et al., 2023).

### Incels’ Approval of Violence

In our study, a substantial proportion of incels felt that violence was “Sometimes” (20%) or “Often” (5%) justified against those who they perceived as seeking to harm them. Although this means that the average response sat between “Never” and “Rarely,” these are still alarmingly high response rates for this measure, supporting research which finds that violence is regularly supported within incel communities (Baele et al., 2023). These findings are also somewhat in line with other incel research; Speckhard et al. (2021) asked participants whether they sometimes entertain thoughts of violence toward others, of which 26% responded affirmatively.

It is difficult to anchor these incel-specific responses to a societal baseline. However, a comparison can be made with Pauwels and Schils (2016), who found that only 2.4% of their sample of 16–24-year-olds expressed some level of support for violent extremism. Their sample is of a broadly similar age range to ours, which is a helpful comparison, but 65% of the respondents were female, which does not help, given the large sex difference in young adult acts of violent crimes (Goodwin et al., 2022; Nivette et al., 2019). At the same time, incels’ endorsement of violence seems comparable to recent nationally representative research, such as that by Wintemute et al. (2022), who found that 20% of the public in the USA thought that violence is sometimes, usually or always justified, while Ipsos (2023) found a similar figure among French respondents.

Finally, we urge caution in over (or under) interpreting findings that relate to the justification of violence in the context of violent extremism. It is an imperfect proxy for radicalization because it measures an attitude rather than behaviors. It likely overestimates the level of individuals who would actually engage in violence because individuals often do not engage in behaviors that they intend to (Conner & Norman, 2022; Sheeran & Webb, 2016). Alternatively, it may underestimate violence because of social desirability bias (Krumpal, 2013).

### What Factors Predict Harmful Attitudes and Beliefs Among Incels?

Good support was found for our predictive model. The core three factors of poor mental health, ideology, and networking predicted harmful attitudes and beliefs, with poor mental health and ideology having twice the predictive power of networking. These two factors also showed covariance—revealing a potential bidirectional effect. These findings support research showing that incel forum-users use of misogynistic terms does not increase with post-frequency, suggesting that members arrive (rather than become) misogynistic (Halpin et al., 2022).

The development and maintenance of incel beliefs may also be explained, in part, by self-verification theory and identity fusion theory (Rousis et al., 2023). Self-verification theory suggests that incels gravitate toward communities that validate their worldview, even when it is negative or harmful. These online spaces likely amplify incels’ sense of rejection sensitivity—validated by their networking experiences—reinforcing their identity as marginalized and misunderstood individuals. This aligns with our findings that networking predicted harmful attitudes and beliefs, as online spaces may provide the “self-verifying” feedback that fuels incel identity.

Identity fusion theory provides additional insight, suggesting that incels with a strong alignment between their personal and group identity may be more likely to endorse extreme attitudes and behaviors to protect or promote the group. This aligns with our observation that ideological adherence was a significant predictor of harmful attitudes and beliefs. Recent research supports this synergistic relationship: incels who feel self-verified by their community are more likely to experience identity fusion, which, in turn, predicts harmful behaviors such as online harassment and endorsement of violence toward women (Rousis et al., 2023). Together, these theories highlight how factors such as bullying, abuse, and low mate value—which push individuals toward online echo chambers—interact with group dynamics to sustain incel ideology and motivate harmful actions.

Except for sociosexuality, all dispositional and experientially developed variables predicted adherence to incel ideology. Poor mental health was predicted by autistic traits, bullying and abuse, dark triad, and low mate value. Finally, increased networking was predicted by bullying and abuse directly, and by autistic traits indirectly via its impact on bullying and abuse.

### The Dual Pathways Hypothesis of Incel Harm

Our findings suggest two distinct pathways to harmful attitudes and beliefs among incels, which we term the dual pathways hypothesis of incel harm. This framework represents an important step in early theory development for understanding the mechanisms driving harmful behaviors in this unique subpopulation. By integrating insights from dispositional traits, psychosocial vulnerabilities, and environmental experiences, this hypothesis offers a structured approach for future research and intervention design.

Pushing previous work forward, our findings offer a highly interesting path analysis, extracting relevant factors—poor mental health, ideology, and engagement with incel networks—that are predictive of harmful attitudes and beliefs. Such an approach begins to impose order on the existing body of research, moving beyond a collection of descriptive facts to propose a cohesive framework that integrates key dispositional and experiential factors. By doing so, we provide a steppingstone for future studies to test, refine, and expand upon these pathways, offering insights that are both theoretically grounded and practically useful. the dual pathways hypothesis of incel harm builds on this foundation by synthesizing past findings and identifying two distinct trajectories that may explain how incel-related harmful attitudes and behaviors may develop.

### Pathway 1: Dispositional Extremism Trajectory

The first pathway involves dispositional traits, such as the Dark Triad (narcissism, psychopathy, and Machiavellianism) and right-wing political views. These traits are associated with heightened misogyny and a greater likelihood of condoning or engaging in violence (Douglass et al., 2023; Jasko et al., 2022; Pailing et al., 2014). Individuals following this pathway may be driven by a sense of entitlement and a propensity for dominance, which aligns with broader patterns of ideological extremism.

### Pathway 2: The Psychosocial Vulnerability Trajectory

The second pathway involves psychosocial vulnerabilities, including autistic traits, low mate value, and adverse childhood experiences such as bullying and abuse. This pathway emphasizes the influence of early life experiences and neurodiversity in shaping harmful coping strategies. These individuals may turn to the black-pill philosophy or hostile online networks as responses to feelings of rejection and isolation.

Although these pathways are conceptually distinct, they are not mutually exclusive. Some individuals may exhibit characteristics or experiences from both pathways, such as possessing traits associated with the Dark Triad while also grappling with psychosocial vulnerabilities like rejection and low mate value. Recognizing this overlap emphasizes the complexity and heterogeneity within the incel community, emphasizing the need for multifaceted and nuanced approaches to research and intervention.

By introducing this hypothesis, we aim to move beyond descriptive studies and begin building a more robust theoretical understanding of the development and maintenance of incel beliefs. The dual pathways hypotheses advances the field by recognizing the heterogeneity within the incel community, challenging overly simplistic narratives that treat incels as a monolithic group. Moreover, this hypothesis emphasizes the importance of understanding how dispositional and experiential factors interact to reinforce harmful beliefs. It echoes findings in related domains, such as radicalization research, which show that interventions are most effective when they address the specific psychological and social drivers of harmful behaviors (e.g., Broyd et al., 2023).

This theoretical framework not only organizes prior findings into a coherent structure but also reveals testable predictions about the heterogeneity within the incel community. As such, it represents an important early step toward theory development in this nascent field, laying the groundwork for future research and practical interventions. In doing so, it aligns with calls for work that goes beyond documenting phenomena to explaining and predicting them (Hart & Huber, 2023).

### Insights for Intervention

Our findings highlight the importance of interventions targeting the psychological and ideological dimensions of incel beliefs, rather than focusing exclusively on networking and the presence of two pathways suggests that interventions must also address both dispositional and experiential pathways to harm.

Targeting Mate Value and Social Skills: Programs focusing on improving men’s self-perceived mate value—such as dating skills training (Li et al., 2020) may reduce the resentment and low self-esteem that often underpin misogyny (Costello et al., 2023). Our model found that low mate value indirectly predicted harmful attitudes and beliefs among incels through its impact on mental health. These results align with prior research linking self-perceived mate value to men's self-esteem (Brase & Dillon, 2022) and findings that men are most misogynistic when they doubt their appeal to women (Bosson et al., 2022) or experience unwanted celibacy (Grunau et al., 2022). Interventions aimed at improving men’s confidence—such as addressing “lack of flirting skills” and “dating anxiety,” which many incels identify as primary barriers (Costello et al., 2023)—show promise in enhancing their dating prospects and reducing harmful attitudes. Similarly, social skills programs for adults with autism have shown promise in improving dating confidence (Płatos et al., 2024). Targeted interventions, such as enhancing dating-app performance—a ubiquitous area of modern dating where incels particularly struggle (Sparks et al., 2024a, 2024b)—might not only improve incels' dating success but, in doing so, mitigate issues like low self-esteem and misogyny. Future research should aim to distinguish between incels’ subjective perceptions of low mate value and their objective mate value, as this distinction has important implications for intervention strategies, particularly regarding self-improvement efforts versus cognitive-behavioral approaches that address distorted self-perceptions.

Addressing Cognitive Distortions and Misperceptions Through Credible Role Models: Correcting incels’ cognitive distortions is another promising intervention target. Due to their rejection sensitivity, incels massively overestimate societal animosity and underestimate sympathy and support for their romantic success (Costello & Thomas, 2025). Only about two-thirds of our sample agreed with our provided ideology definition, indicating some fragility to this belief that could be reasonably deconstructed by credible messengers (Ellefsen & Sandberg, 2022).

Incels identified feminists as their primary enemy, and in other research, feminist identification was associated with greater blame, reduced sympathy, and heightened animosity for incels, particularly among women. Although some scholars advocate for feminist-led approaches to incel intervention (see Costello et al., 2024a), the mutual animosity between incels and feminists raises questions about the efficacy of such efforts. Evidence suggests that deradicalization is most effective when led by individuals seen as “credible insiders” (Ellefsen & Sandberg, 2022; cf. Braddock, 2022; Koehler et al., 2023). Former incels who have disengaged from these communities, such as members of the r/IncelExit subreddit may serve as more effective role models (Burns & Boislard, 2024; Thorburn, 2023).

Leveraging Emerging Technologies: There are several technological solutions that can be used to aid interventions. To find and successfully target incels, AdWord technologies can be used in conjunction with incel lexicon to divert them to interventions, as has been done previously with jihadist and far-right content (Moonshot, 2017, 2020). Once incels have been identified and diverted, AI-based chatbox technologies can play a role in interventions. Presently, several bespoke chatboxes are being designed to draw from existing debates to craft persuasive arguments (Vallecillo-Rodríguez et al., 2023). In one study brief conversations with GPT-4 have been shown to reduce entrenched conspiracy beliefs by 20% (Costello et al., 2024b) and could be adapted to address incels’ black-pill philosophy. This could, in particular, be useful for those on Pathway 1, who display right wing political beliefs (Dyrendal et al., 2021) and the Dark Triad (Došenović & Dinić, 2024; Hughes & Machan, 2021), which are often linked to conspiracy beliefs. We should urge caution here: We do not believe that AI is the solution to extremist beliefs or violence, but the 24-h availability of a chatbox could be a useful first part of an intervention, to be used in conjunction with professionals.

Reducing Isolation and Strengthening Real-World Connections: Loneliness is a well-documented issue among incels (Costello et al., 2022); for example, a 2018 incels.co poll found only one-third reported having friends (Jeltsen, 2018). This isolation likely fuels misperceptions, as incels rely on nihilistic online echo chambers which may reinforce their distorted views (Costello et al., 2024a). Networking with other incels is also linked to displaced aggression and rumination. Finally, forum use has also been shown to predict depression among incels, and many report that their opinions of women have worsened since joining these online communities (Costello et al., 2022). Promoting friendships outside of toxic online spaces could mitigate at least some of these distortions. Real-world connections may reduce the reliance on online echo chambers that reinforce incels’ bleak worldview.

Taken together, our findings emphasize that multifaceted psychosocial interventions focusing on mental health and ideology may be more effective than those targeting networking, although all are important. Although incel research is still in its infancy, there is now enough foundational research to inform the design and testing of some different tailored interventions to establish which have efficacy.

### Limitations and Future Directions

This study had some limitations. First, the cross-sectional nature of this study makes it harder to determine whether poor mental health, ideology, or networking precede or result from incel identification. Longitudinal research exploring the long-term trajectories of incels is needed to inform potential deradicalization pathways.

Incel research is in its infancy, and this study reflects some of the challenges inherent to studying a nascent and evolving phenomenon. One such limitation is our measurement of ideology. While our composite measure was constructed based on commonly endorsed beliefs among incels identified in the existing literature, we are not aware of any well validated scale of adherence to incel ideology that currently exists. We recognize this as a limitation and hope that our empirical findings contribute toward the development and validation of such a scale in the future. Until then, our approach should be seen as a preliminary effort to quantify ideology rather than a definitive measure.

Additionally, our recruitment strategy, though rigorous in screening, may have introduced bias. By intentionally targeting known incel communities, we ensured that our sample included individuals most likely to identify with the subculture. However, this approach may exclude individuals on the periphery of these communities or those who are hesitant to engage with researchers. Furthermore, given the widespread suspicion of researchers within incel forums, biased responding remains a possibility. To mitigate this, we employed a rigorous screening process to ensure data quality and participant authenticity (see Method section for details).

Another limitation is the reliance on self-report measures, which may not fully capture real-world hostility, aggression, or violence. While these measures provide valuable insights into participants’ attitudes and beliefs (Corneille & Gawronski, 2024), future research should incorporate behavioral measures or longitudinal designs to better assess how these attitudes manifest in real-world actions. The most comprehensive understanding of the incel phenomenon will result from triangulating primary data such as ours with other forms of research (e.g., linguistic analyses of forum content and qualitative research).

Finally, although we found few differences between the UK and US subsamples, our WEIRD (Western Educated Industrialized Rich and Democratic) sample (Henrich et al., 2010) limits the generalizability of our findings. Wider cross-cultural research may offer insights into the diversity of incel experiences, the impact of different mating ecologies (Brooks et al., 2022), and intervention efficacy across settings.

### Conclusion

This study represents a critical step forward in understanding the psychological, ideological, and social factors that contribute to harmful attitudes and beliefs within the incel community. Inspired by the 3N framework (Kruglanski et al., 2019), we found that the three core factors of poor mental health, ideology, and networking predicted incel harm both individually and, in some cases, in tandem. Furthermore, a consideration of dispositional and experiential antecedents revealed two distinct pathways to incel harm: Dispositional Extremism and Psychosocial Vulnerability. These pathways form the basis of our newly proposed dual pathways hypothesis of incel harm, which provides a nuanced perspective on the different ways some incels may form harmful attitudes and beliefs.

Our findings highlight the interactive relationship between poor mental health, ideological adherence, and networking behaviors, emphasizing the need for multifaceted psychosocial interventions. These interventions must address not only the dispositional traits and adverse experiences that predispose individuals to incel beliefs but also the cognitive distortions and societal misperceptions that perpetuate them.

Importantly, our study emphasizes the heterogeneity within the incel community, revealing distinct pathways to harmful attitudes and beliefs that demand tailored intervention strategies. This diversity presents both challenges and opportunities for researchers and practitioners, as it necessitates a careful balance between addressing commonalities within the group and accommodating the unique needs of its subgroups, such as incels who may have autism.

Incel research remains a nascent and evolving field. However, the growing body of literature, including the findings from this study, offers a promising foundation for the development of evidence-based interventions. Future research should prioritize longitudinal designs, the validation of incel-specific ideological measures, and the exploration of behavioral outcomes to better understand the trajectories and real-world impacts of incel beliefs. Our study sets a new benchmark in incel research, emphasizing the importance of addressing mental health challenges and ideological beliefs in this hard-to-reach community of men. This foundational research can now inform the design of targeted interventions to address the challenges incels face and the issues they represent to society.

## Supplementary Information

Below is the link to the electronic supplementary material.

Supplementary file1 (DOCX 37 KB)

## Data Availability

There are restrictions on data availability due to ethical considerations and participant privacy. Inquiries regarding data availability should be directed to the corresponding author.

## Footnotes

1 These participants also agreed that they were incel; thus, this “reason” likely captures the sentiment held by many incels that they are voluntarily opting out of mating due to their hopeless prospects.

## Code Availability

Available upon request to the corresponding author.
