# Supplementary material for: The Dual Pathways Hypothesis of Incel Harm: A Model of Harmful Attitudes and Beliefs Among Involuntary Celibates
Source: Arch Sex Behav. 2025 May 21;54(5):1815–36. doi: 10.1007/s10508-025-03161-y (PMC12162687; doi:10.1007/s10508-025-03161-y)
Supplement: Supplementary file 1 — Supplementary file1 (DOCX 37 KB) [file 10508_2025_3161_MOESM1_ESM.docx]

**Supplementary materials**

**Table S1.
*Cross-cultural differences for the political orientation, mental health, ideological adherence, and networking measures.***

|  | | UK | US | *t* / χ2 (df) | *p* | *d* / *V* |
| --- | --- | --- | --- | --- | --- | --- |
| Political belief | |  |  |  |  |  |
|  | Environmental regulations hurt economy | 100 (50.3%) | 143 (39.5%) | 6.04 (1) | .01 | .10 |
| Depression | |  |  |  |  |  |
|  | Loneliness, mean (SD) | 2.43 (0.64) | 2.59 (0.51) | 3.27 (559) | < .01 | 0.29 |
| Enemies | |  |  |  |  |  |
|  | Incels themselves, mean (*SD*) | 2.87 (1.36) | 3.16 (1.38) | 2.38 (559) | .02 | 0.21 |
| Anonymous forums | |  |  |  |  |  |
|  | Radical people | 2.79 (1.10) | 3.31 (1.06) | 3.99 (306) | < .001 | 0.48 |
|  | Radical content | 2.79 (1.12) | 3.26 (1.14) | 3.46 (306) | < .001 | 0.41 |
|  | Feelings of support | 3.66 (0.96) | 3.32 (0.87) | 3.06 | < .01 | 0.37 |
| Registered forums | |  |  |  |  |  |
|  | Often used (over 25%) | 54 (27.1%) | 137 (37.8%) | 6.56 (1) | .01 | .11 |
|  | Primary (over 50%) | 42 (21.1%) | 107 (29.6%) | 4.70 (1) | .04 | .09 |
|  | Radical people | 2.51 (1.08) | 2.81 (1.11) | 2.18 (284) | .03 | 0.28 |
| In Person | |  |  |  |  |  |
|  | Often used (over 25%) | 18 (9.0%) | 17 (4.7%) | 4.15 (1) | .04 | .09 |
| Video calls | |  |  |  |  |  |
|  | Ever used | 19 (9.5%) | 17 (4.7%) | 5.03 (1) | .03 | .10 |
|  | Often used (over 25%) | 5 (2.5%) | 2 (0.6%) | 4.00 (1) | < .05 | .08 |
| Messaging apps | |  |  |  |  |  |
|  | Ever used | 52 (26.1%) | 47 (13.0%) | 15.27 (1) | < .001 | .16 |
|  | Primary (over 50%) | 13 (6.5%) | 7 (1.9%) | 7.90 (1) | < .01 | .12 |
|  | Feelings of support | 3.86 (0.70) | 3.46 (0.76) | 2.73 (97) | < .01 | 0.55 |
| Average across networks | |  |  |  |  |  |
|  | Radical people | 2.56 (0.97) | 2.84 (1.06) | 2.86 (440) | < .01 | 0.28 |
|  | Radical content | 2.52 (0.99) | 2.81 (1.00) | 2.89 (440) | < .01 | 0.29 |
|  | Feelings of support | 3.58 (0.82) | 3.28 (0.81) | 3.71 (440) | < .001 | 0.37 |

**Table S2.
*Results of a principal components analysis to produce Poor mental health, Ideological adherence, Networking, and Harmful attitudes and beliefs variables.***

| Trait (Variance Explained) | | Loading | KMO | Bartlett's |
| --- | --- | --- | --- | --- |
| Poor mental health (58.5%) | |  | 0.70 | χ^2^(6) = 641.56, *p* < .001 |
|  | PHQ-9 | 0.86 |  |  |
|  | GAD-7 | 0.81 |  |  |
|  | Loneliness | 0.69 |  |  |
|  | Rejection sensitivity | 0.67 |  |  |
| Ideological adherence (39.2%) | |  | 0.74 | χ^2^(21) = 810.36, *p* < .001 |
|  | Feminists (enemies) | 0.74 |  |  |
|  | Women (enemies) | 0.74 |  |  |
|  | Wider society (enemies) | 0.69 |  |  |
|  | The political left (enemies) | 0.64 |  |  |
|  | Feelings of discrimination | 0.65 |  |  |
|  | Belief in ideology (80/20 rule) | 0.44 |  |  |
|  | Belief in a shared incel worldview | 0.38 |  |  |
| Networking in a typical week (65.4%) | |  | 0.84 | χ^2^(21) = 2393.99, *p* < .001 |
|  | Exposure to radical people | 0.90 |  |  |
|  | Exposure to radical content | 0.89 |  |  |
|  | Time spent | 0.87 |  |  |
|  | Number networks used | 0.85 |  |  |
|  | Number of incels engaged | 0.84 |  |  |
|  | Frequency of interaction (vs lurking) | 0.64 |  |  |
| Harmful attitudes and beliefs (48.7%) | |  | 0.72 | χ^2^(15) = 1108.86, *p* < .001 |
|  | Revenge Planning | 0.86 |  |  |
|  | Angry Rumination | 0.73 |  |  |
|  | Hostile Sexism | 0.68 |  |  |
|  | Rape Myth Acceptance | 0.67 |  |  |
|  | Displaced Aggression | 0.64 |  |  |
|  | Violence, mean (*SD*) | 0.57 |  |  |
